# *In Vitro* Infection of *Trypanosoma cruzi* Causes Decrease in Glucose Transporter Protein-1 (GLUT1) Expression in Explants of Human Placental Villi Cultured under Normal and High Glucose Concentrations

**DOI:** 10.1155/2012/969243

**Published:** 2011-09-15

**Authors:** Luciana Mezzano, Gastón Repossi, Ricardo E. Fretes, Susana Lin, María José Sartori, Sofía G. Parisi de Fabro

**Affiliations:** ^1^Cátedra de Biología Celular, Histología y Embriología, Instituto de Biología Celular, Facultad de Ciencias Médicas, Universidad Nacional de Córdoba, Ciudad Universitaria, 5016 Córdoba, Argentina; ^2^Cátedra de Histología, Embriología y Genética, IICSHUM, Universidad Nacional de La Rioja, 5300 La Rioja, Argentina; ^3^Division of Environmental Health and Occupational Medicine, National Health Research Institutes, 35053 Zhunan Town, Miaoli County, Taiwan

## Abstract

*Trypanosoma cruzi*, the etiologic Chagas' disease agent, induces changes in protein pattern of the human placenta syncytiotrophoblast. The glucose transporter protein-1 (GLUT1) is the primary isoform involved in transplacental glucose transport. We carried out *in vitro* assays to determine if *T. cruzi* infection would induce changes in placental GLUT1 protein expression under normal and high concentration of glucose. Using Western blot and immunohistological techniques, GLUT1 expression was determined in normal placental villi cultured under normal or high concentrations of glucose, with or without *in vitro T. cruzi* infection, for 24 and 48 hours. High glucose media or *T. cruzi* infection alone reduced GLUT1 expression. A yet more accentuated reduction was observed when infection and high glucose condition took place together. We inform, for the first time, that *T. cruzi* infection may induce reduction of GLUT1 expression under normal and high glucose concentrations, and this effect is synergic to high glucose concentrations.

## 1. Introduction

Chagas' disease, endemic in Latin America, is caused by *Trypanosoma cruzi* a flagellated protozoan with a life cycle involving an insect vector and a mammalian host. The congenital Chagas' disease is associated with premature labor, miscarriage, and placentitis [1]. In endemic countries, maternal-fetal transmission of *T. cruzi* is between 1 and 17% of pregnancies in chronically infected mothers, depending on geographic area [2]. The infectious form of the parasite (trypomastigote) adheres to specific receptors on the outer membrane of host cells previous to intracellular invasion [3], and the process of invasion requires activation of signal transduction pathways in the parasite and the host cell [4–10].

Pancreas is one of the organs affected in Chagas' Disease. Patients with this disease have plasma pancreatic glucagon and pancreatic polypeptide levels reduced [11], lower insulin activity [12], and morphometric and morphologic alterations of pancreatic ganglia and islets [13]. Experimental infections in hamsters caused pancreatitis, erratic blood glucose levels, and a tendency to hypoinsulinemia [14]. *T. cruzi *infection-resistant C57BL/6 mice developed high parasitemia and mortality when diabetes was induced with streptozotocin in them [15]. Above clinical and animal studies suggest that patients with Chagas' Disease are more susceptible to develop hyperglycemia, and this would worsen the infection. In fact, patients with both conditions have been reported, and diabetes or hyperglycemia prevalence was higher in patients with the cardiac form of Chagas' disease [16].

Hyperglycemia during pregnancy is a well-recognized pathogenic factor. Adequate glucose transfer from the maternal to the fetal compartment is crucial for the normal survival and development of the fetus during pregnancy [17]. GLUT1 is the primary isoform involved in the transplacental movement of glucose and distributed asymmetrically on the microvillus and basal membranes syncytiotrophoblast. The microvillous membrane contains more transporters than the basal. GLUT is inversely regulated by glucose concentration, and basal membrane GLUT1 is positively regulated by insulin-like growth factor I, placental growth hormone, and hypoxia [18]. *In vivo*, basal membrane GLUT1 is upregulated over gestation, increased in diabetic pregnancy, and decreased in chronic hypoxia, while microvillous membrane GLUT1 is unaffected [18, 19]. As the rate-limiting step in transplacental glucose transport, changes in the density of basal membrane GLUT1 will have a significant impact on transplacental glucose flux [18, 20].

Although increased expression of GLUT1 in placenta from diabetic pregnant women has been reported [18, 20], decrease in GLUT1 mRNA and protein levels in diabetic mice compared with the control and placental cell cultures under high glucose concentration conditions was also informed [20, 21]. Glucose would also alter GLUT1 partitioning between the plasma membrane and intracellular sites in favor of the latter [22]. 

In the invasion process, *T. cruzi *has been found to affect numerous surface molecules of the placental villi, probably causing placental dysfunction as consequence. We wondered if this parasite would somehow also affect GLUT1 expression pattern, especially under high glucose (HG) concentration, since *T. cruzi *has been reported to affect pancreatic function and alters the insulin-glycemia axis, and there is not yet a marker for placental or fetus infection. In this work, we compared the protein expression of GLUT1 of human placental explants infected *in vitro* with trypomastigotes, cultured under normal and high glucose (HG) concentration.

## 2. Materials and Methods

### 2.1. Placentas

Placentas (*n* = 17) from clinically and serologically healthy women at 38 to 40 weeks of gestation were obtained by caesarian delivery, in order to assure asepsis. Women signed an informed consent. Placentas were kept in glucose solution 0.29 mM at 4°C for transportation. Once in the laboratory, central villi of placental cotyledons were isolated, rinsed with PBS several times, and cut into pieces of 3 mm in diameter.

### 2.2. Parasites

Trypomastigotes of *T. cruzi *(Tulahuen strain) were isolated according to Andrews and Colli [23] and Fretes and Fabro [24], from infected Albino/Swiss mice bloodstream at the peak of parasitism. Briefly, mice blood was centrifuged for 10 minutes at 100 g and kept still for 1 h at 37°C. Plasma was then separated and centrifuged for 10 minutes at 590 g. The pellets containing the parasites were washed twice and suspended in MEM-199 (Gibco Lab., NY, USA).

### 2.3. Treatments of Placental Explants

Explants of normal human placental villi were cultured at 37°C, in normal atmosphere supplemented with 5% CO_2_, in MEM-199 (pH 7) with 0.1% penicillin and 0.01% streptomycin and either with 5 mM D-glucose (normal D-glucose: NG) (J.T. Baker, NJ, USA) or 25 mM D-glucose (high glucose: HG) concentrations [22]. 

After 24 hours, 7 × 10^5^ trypomastigotes Tulahuen strain of *T. cruzi* were added with the refresh of culture media, prepared as mentioned above. Controls without parasites were carried out for both normal and HG concentrations. Cocultures with trypomastigotes and controls were incubated for another 24 h or 48 h.

After treatment, the placental explants were rinsed with PBS. Part of the explants were fixed with 10% formaldehyde and included in paraffin. Approximately 30 mg of placental explants from each treatment were homogenized in 0.25 mL PBS with an OMNI 1000 homogenizer for five cycles of high speed application, each lasting ten seconds. 

### 2.4. Analysis of Placental Explants Infection

At the end of cultures, placental explants were collected, fixed, and stained with hematoxylin/eosin and PAS/hematoxylin and observed under low and high magnifications in a Zeiss Axioskop 20 microscope. Infection of placental explants was assessed observing amastigote groups of the *T. cruzi* in trophoblast or stromal cells of the chorionic villi. 

### 2.5. Immunostaining of GLUT1 Protein Expression in Placental Explants

Deparaffinized histological sections were embedded in TBS (pH 7.2), pretreated with 0.05% saponin (15 min) for the unmasking of antigens, then with 3% H_2_O_2_ (15 min) to block internal peroxidases, and treated with 3% nonfat dry milk in TBS (15 min) to block nonspecific epitopes. GLUT1 protein was detected by incubating the treated sections with an anti-GLUT1 polyclonal antiserum (rabbit, CHEMICOM International Inc, Temecula, Calif, USA) diluted in TBS/Tween (1 : 500) for overnight, at 4°C, and revealed with a secondary antirabbit immunoglobulin conjugated with peroxidase (Sigma-Aldrich Co, Mo, USA). Peroxidase activity was developed using H_2_O_2_/4-Cl-1-naphthol. Background control without addition of anti-GLUT1 antiserum was carried out. We also used precultured placenta controls. Images stored as jpg format were analyzed with “Image Tool” UTHSCSA version 3.00 (downloadable from http://ddsdx.uthscsa.edu/dig/download.html) as described previously [25]. Five optical fields were measured per slide; each treatment for each placenta provided 3 slides. Immunostained area was expressed as ratio of total area (manually selected surface area).

### 2.6. Western Blot for GLUT1

Homogenized placental villi were mixed with lysis buffer containing protease inhibitors (1% w/v de SDS, 1 mM EDTA, 1 *μ*g/mL of leupeptin, 100 mM of Hepes pH 7.4) and centrifuged at 12000 g for 10 minutes. Protein content was measured with Lowry protein assay [26]. The samples were not heated prior electrophoresis; 40 *μ*g of sample were loaded per lane in a SDS-PAGE gel that was carried out with 3% stacking gel and 10% resolving gel at 200V for 1 hr [27] and blotted onto nitrocellulose at 300 mA for 1.5 hr using a Trans Blot Mini-Protean II apparatus (BioRad, Richmond, Calif, USA). GLUT1 on nitrocellulose was detected with rabbit anti-GLUT1 polyclonal antiserum (CHEMICOM International Inc, Temecula, Calif, USA), diluted 1 : 5000 in TBS-Tween and revealed with a secondary antirabbit immunoglobulin conjugated with peroxidase (Sigma-Aldrich Co, Mo, USA). Peroxidase activity was developed using H2O2/4-Cl-1-naphthol. Background control without addition of anti-GLUT1 antiserum and precultured placenta controls were carried out. Actin was detected as a positive control for protein content present in samples, due the stable expression of this protein.

GLUT1 expression was evaluated with the “Scion image for windows” program (version: Beta 4.0.2) to measure the area units marked by Western blot. 

### 2.7. Statistical Analysis

Data were expressed as the mean ± SE and were analyzed statistically by ANOVA test followed by “post hoc” LSD Fisher test. Paired *t*-test was performed, to establish significance between groups (different placentas). A level of less than 5% (*P* < 0.05) was chosen to detect significant differences. 

## 3. Results

**Figure 1:**
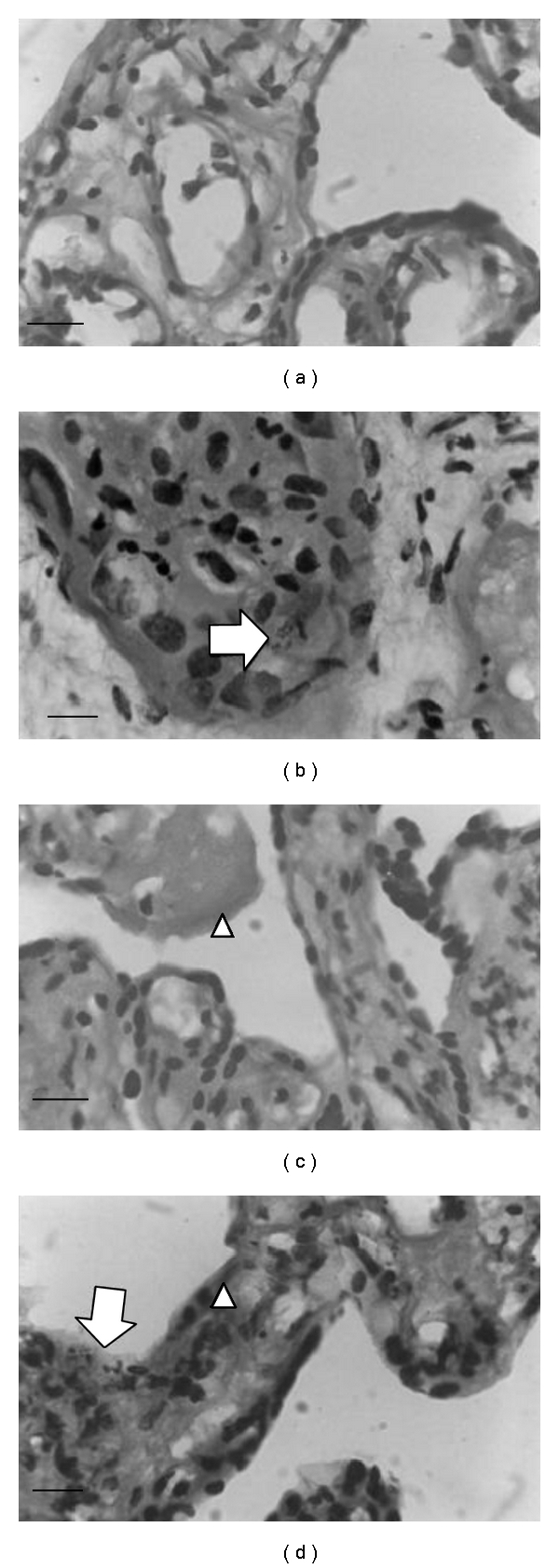
Paraffin-embedded placental villi cultured for 48 hs, Pas/H stained. (a) Control placental villi without *T. cruzi* infection cultured with normal glucose concentration media; (b) normal placental villi *in vitro *infected with *T. cruzi,* cultured with normal glucose media; (c) normal placental villi cultured under high glucose concentration media; (d) normal placental villi cultured under high glucose concentration media, *in vitro *infected with *T. cruzi.* Amastigotes nests were observed inside the villous from infected placentas (arrows). Arrow points indicate increased glycogen deposits in placental villi explants cultured under HG concentration (c and d). Original magnification: 1000x. The scale bar represents 1 *μ*m.

Placental explants showed groups of the reproductive forms of the parasite (amastigotes) mainly in stromal cells of the chorionic villi under normal (NG) and high D-glucose (HG) concentrations, as they were seen in placental villi slides stained with PAS/H. Furthermore, the presence of the parasite was more evident when placental explants were cultured with high D-glucose concentration (Figure 1). 

Placental villi explants cultured under HG concentration (HGC) underwent structural modifications mainly thickening of the syncytiotrophoblast and basal membranes, increased glycogen deposits, and a fetal capillary thickening, similar to those described in the bibliography [28–30]. These alterations were maintained and also were more notorious when these placental explants were infected with *T. cruzi*, compared to healthy controls incubated with normal glucose concentration (NGC), as shown in placental sections with PAS/H staining (Figure 1).

**Figure 2:**
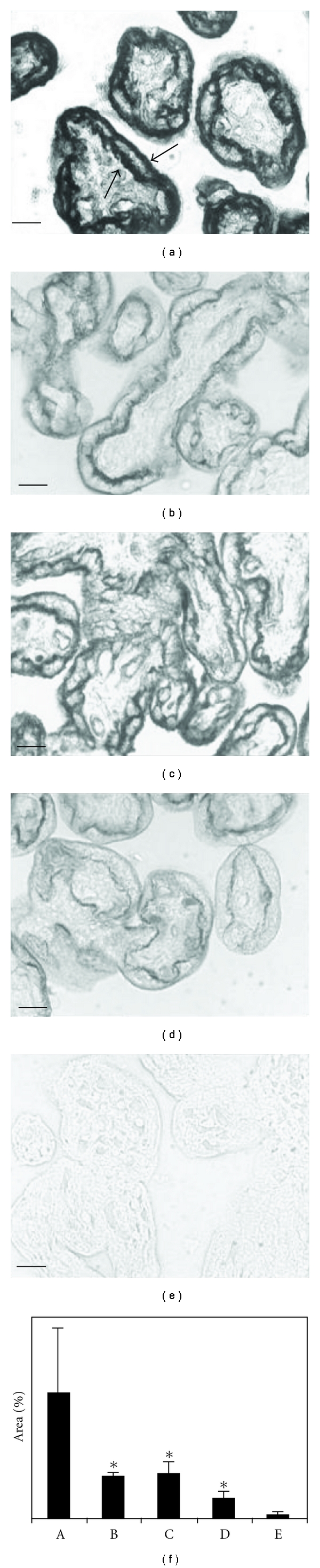
GLUT1 immunodetection in paraffin-embedded placental villi sections cultured for 24 hours. (a) Uninfected control placental villi. GLUT1 expression was intensively marked on the apical and basal syncytiotrophoblast cell membrane (arrows); (b) normal placental villi *in vitro *infected with *T. cruzi*; (c) normal placental villi cultured under high glucose concentration media; (d) normal placental villi cultured under high glucose concentration media, and *in vitro *infected with *T. cruzi*; (e) negative control placental villi (without GLUT1 antiserum incubation); (f) image quantification made by “Image Tool” UTHSCSA version 3.00, which indicates the percentage of the marked area; values shown are the mean ± SE of four samples from representative experiments (A: control placental villi, without infection; B: infected normal placental villi; C: normal placental villi cultured under high glucose media; D: infected normal placental villi cultured under high glucose media; E: negative control placental villi). Original magnification: 400x. The scale bar represents 1 *μ*m. *Significantly different from the control placental villi (*P* < 0.05; *n* = 4; paired *t*-test).

**Figure 3:**
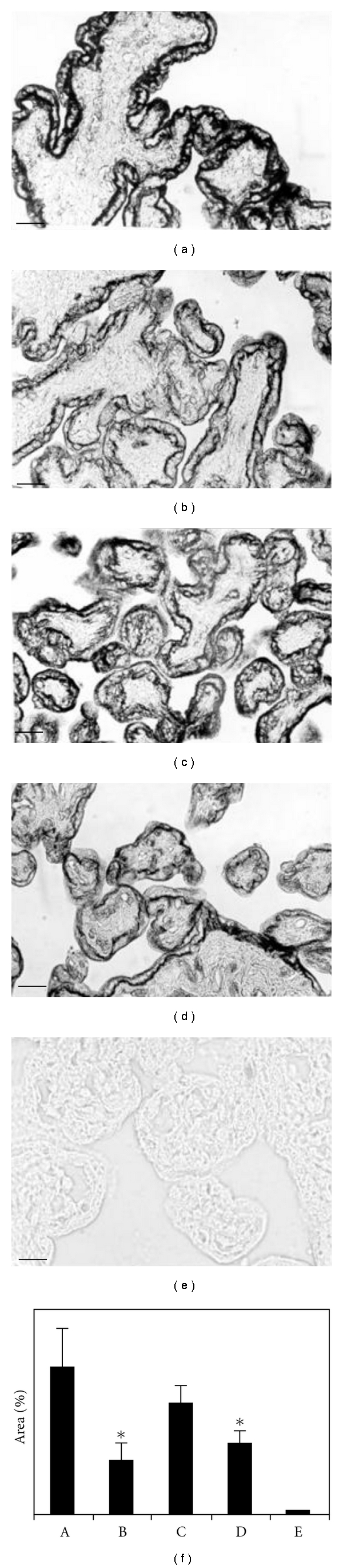
GLUT1 immunodetection in paraffin-embedded placental villi sections cultured for 48 hours. (a) Uninfected control placental villi (GLUT1 expression was intensively marked on the apical and basal syncytiotrophoblast cell membrane); (b) normal placental villi *in vitro *infected with *T. cruzi*; (c) normal placental villi cultured under high glucose concentration media; (d) normal placental villi cultured under high glucose concentration media and *in vitro *infected with *T. cruzi*; (e) negative control placental villi (without GLUT1 antiserum incubation); (f) image quantification made by “Image Tool” UTHSCSA version 3.00, which indicates the percentage of the marked area; values shown are the mean ± SE of seven samples from representative experiments (A: control placental villi, without infection; B: infected normal placental villi; C: normal placental villi cultured under high glucose media; D: infected normal placental villi cultured under high glucose concentration media; E: negative control placental villi). Original magnification: 400x. The scale bar represents 1 *μ*m.*Significantly different from the control placental villi (*P* < 0.05; *n* = 7; paired *t*-test).

GLUT1 protein was intensively stained on the apical and basal membrane of the syncytiotrophoblast in controls with NGC and no infection. The apical expression of GLUT1 was scarce under HGC, infection with *T. cruzi*, or both conditions together, even thought the label increased in basal area under these conditions (Figures 2 and 3).

Image quantification of immunostained areas showed that placental explants cultured under HGC *per se *reduced the expression of GLUT1 to 34.7% at 24 h (Figure 2) and the level of GLUT1 protein expression recovered to 76.3% at 48 h (Figure 3). In explants invaded by *T. cruzi*, the expression of GLUT1 protein was reduced under both NG and HG concentrations. At 24 h, the protein expression was reduced to 32% under NGC and to 14.8% under HGC. At 48 h, the GLUT1 protein expression recovered to 37.4% of the initial levels under NGC and to 48.6% under HGC (paired *t*-test, *P* < 0.05).

**Figure 4:**
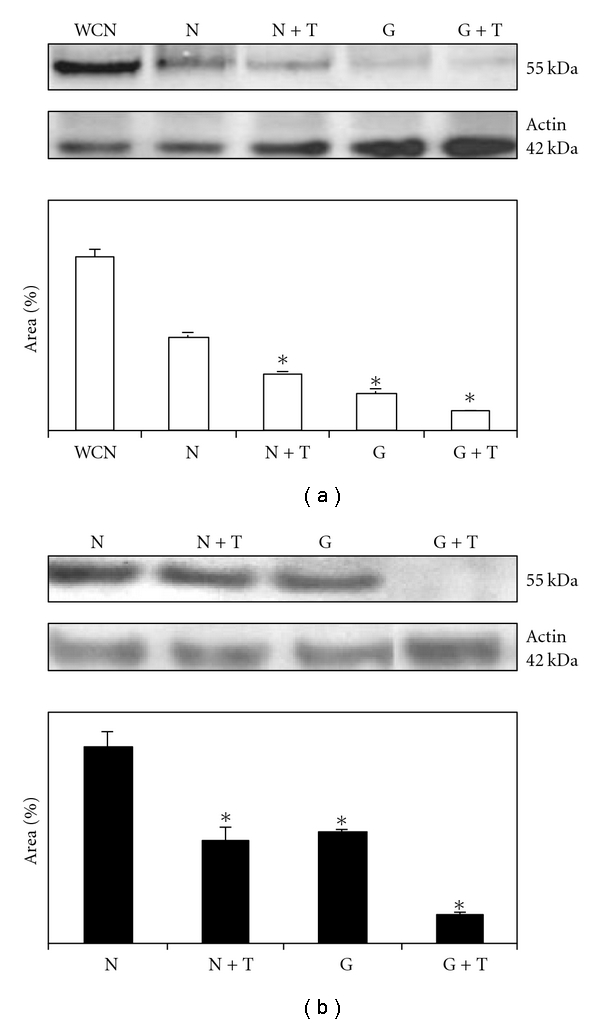
Western blot to detect GLUT1 transporter protein expression in homogenates from normal placental villi, cultured for 24 hours (a) and 48 hours (b). Actin presence was also detected as a positive control for protein content in samples. GLUT1 expression was evaluated with the “Scion Image for Windows” program that measured the area units marked. WCN: without culture normal placental villi (positive control); N: normal placental villi; N + T: normal placental villi cocultured with *T. cruzi; *G: normal placental villi cultured under high glucose media; G + T: normal placental villi cocultured with *T. cruzi *under high glucose media. Values shown are the mean ± SE. *Significantly different from the control (*P* < 0.05; *n* = 8; ANOVA test).

Western-blotting image of one of the placental samples as representative of the experiment is shown in Figure 4. Placental explants cultured under HGC *per se *reduced the expression of GLUT1 to 40.5% at 24 h and the level of GLUT1 protein expression recovered to 56.5% at 48 h. In explants cultured with *T. cruzi*, the expression of GLUT1 protein was reduced under both NG and HG concentrations. At 24 h, the protein expression was reduced to 59.5% under NGC and to 20% under HGC. At 48 h, the GLUT1 protein expression was at 52.1% and 14.85% of the initial levels, in cultures under NG and HG concentrations, respectively (*P* < 0.05).

## 4. Discussion

Different authors have reported that diabetics have an increased susceptibility to a variety of infectious agents [31, 32]. Hyperglycemia has been previously observed to increase the morbidity and mortality of murine *T. cruzi *infection [15]. Diabetes and hyperglycemia were also reported to be more prevalent in chagasic human patients with the cardiac form of the disease, than in control ones [16]. But, according to the analysis of the bibliography, there is not any study analyzing an association between pregnant women affected with both Chagas' disease and diabetes with congenital transmission or with the effect on the new born. Furthermore, there is no marker for placental or fetus infection in Chagas' disease. Due the effect of the Chagas parasite on some proteins located at the lipid raft of chorionic villi trophoblast [24, 33], as well as in trophoblast lipids [34], we aimed to analyze the possible modification of the main glucose transporter located at the syncytiotrophoblast, the GLUT1 protein, produced by placental *T. cruzi* infection. 

*T. cruzi *crosses the placental barrier and infects the fetus, causing the congenital form of the disease [1]. In order to understand the mechanism used by the parasite to cross this barrier, the interaction between syncytiotrophoblast plasma membranes from the human placenta and the parasite was previously studied, with modifications in lipid and protein patterns from trophoblast membranes being found [35]. However, the mechanism by which the parasite infects the placenta as well as the effects upon the protein contents of the placental barrier is still not well understood.

Our experiments showed that GLUT1 protein expression was significantly diminished in normal placental villi cultured under HGC *in vitro *infected with *T. cruzi, *compared to controls, as was observed by GLUT1 immunodetection and western blot. Previous studies have shown that the downregulation of GLUT1 mRNA expression is correlated with decreased glucose uptake in placental trophoblasts cultured under high D-glucose concentration [22]. In the present work, a significant reduction in GLUT1 protein expression was observed in normal placental syncytiotrophoblast, cultured under HGC for either 24 or 48 hours. This corroborates the inverse relation existing between GLUT1 expression and extracellular glucose concentration, as described by various authors [18, 20, 36]. Our results are consistent with previous observations that a reduction in mRNA expression of GLUT1 is induced by a high glucose concentration in cultured placental trophoblast cells [22, 36–38].

*T. cruzi *produces plasmatic membrane modifications, by altering their lipid [34, 35] and protein components, such as placental alkaline phosphatase [7–9, 39–41] and GLUT1 protein, as observed in the present study. On the other hand, we observed in our laboratory, that Gamma-glutamyl transpeptidase, another enzyme present in the placenta's brush border, is not affected in cells cocultured with *T. cruzi *[33], suggesting that the parasite affects molecules inserted in lipid microdomains of the membrane, as PLAP [42] and GLUT1 [43]. As the hyperglycemia characteristic of diabetes mellitus also affects these membrane components, we suggest that both the parasite and the high glucose conditions could have been provoking, by different ways, a lower placenta efficacy transporting glucose to fetus. The results obtained in this work demonstrate that placental explants infected with *T. cruzi *induce changes in GLUT1 expression from human term placenta under high D-glucose concentration. As a consequence, it is of importance to perform a systematic study analyzing new born from pregnant women who have both conditions diabetes and Chagas. 

Reduced GLUT1 expression observed in placental culture with parasites could imply a downregulated GLUT1 activity in pregnant women with Chagas' disease. If this disease is causing damage to pancreas islets [11–13], or happens together with a previous diabetic condition, the adverse effects of reduced GLUT1 activity may be exacerbated. The level of GLUT1 is regulated by glucose concentration, insulin-like growth factor I, placental growth hormone, and hypoxia [18]. It would be interesting to further study the mechanisms by which GLUT1 is affected in Chagas' disease. It is likely that the parasite would perturb other components of the maternal and fetal glucose-insulin-GLUT1 axis. As glucose and GLUT are inversely regulated, changes in GLUT expression might alter the insulin level in plasma, pointing out to determine a marker for placental infection by *T. cruzi* in pregnant chagasic women. This matter could be of the upmost importance in congenital Chagas' disease.

Histological studies have demonstrated that placentas from poorly controlled diabetic pregnancies show a thickening of the basal membranes and reduced vascularization of the villi [30]. Similar to observations reported by other authors [28, 29], in the present work we detected that placental villi cultured under high glucose conditions were morphologically altered, with thickening of the basement membranes. The presence of *T. cruzi* maintains this alteration. The parasite causes disorganization of the basal membrane of the trophoblast and the stroma of the chorionic villi [44], as was observed in *in vitro* experiments similar to those performed in this work. So, both conditions hyperglycemia and *T. cruzi* can modify the stroma of the chorionic villi. According to our results, the alteration of the trophoblast basal membrane in the presence of *T. cruzi* under high D-glucose concentration might be caused by two different mechanisms or not. This important matter should be elucidated.

The protein membrane pattern was also altered, as noted in others works on Placental Alkaline Phosphatase (PLAP) [7–9, 24, 25, 39–41]; and in the present study with GLUT1 protein. These characteristics could modify the human placenta efficacy as a barrier against infections. It has been described that chorionic villi *in vitro* has low susceptibility to infection by *T. cruzi* in normal D-glucose concentration [45, 46]. In order to clarify if high D-glucose concentration can increase the infection of chorionic villi by *T. cruzi*, it is necessary to quantify the infection by *T. cruzi* of the placental explants under high glucose concentration, in order to analyze the susceptibility of the invasion and the reproduction of the parasite in the placenta, aspect that we are planning to do in further experiments.

With the present work, we report for the first time the effect of *T. cruzi *on GLUT1 protein expression, adding it to the increasing list of affected proteins altered by this parasite [7–9, 24, 25, 40, 41].
